# Histoanatomic and Quantitative Substaging of Lamina Propria Invasion in pT1 Bladder Urothelial Carcinoma: Associations with Recurrence and Progression

**DOI:** 10.3390/diagnostics16213605

**Published:** 2026-11-01

**Authors:** Tuğçem Bıçak, Yekta Bıçak

**Affiliations:** 1 Department of Pathology, University of Health Sciences, Gazi Yaşargil Training and Research Hospital, 21070 Diyarbakır, Türkiye; 2 Department of Urology, University of Health Sciences, Gazi Yaşargil Training and Research Hospital, 21070 Diyarbakır, Türkiye

**Keywords:** bladder cancer, urothelial carcinoma, pT1, lamina propria invasion, substaging, progression

## Abstract

**Objectives**: This study aimed to evaluate lamina propria invasion in pT1 bladder urothelial carcinoma using different substaging methods and to compare the relationships of these methods with recurrence and progression. **Methods**: In 140 retrospectively evaluated cases of pT1 urothelial carcinoma, muscularis mucosae-based histoanatomic substaging, maximum invasion depth, maximum horizontal invasion width, number of invasive foci, and T1m/T1e-based micrometric classifications were examined. **Results**: Recurrence was detected in 55 patients (39.3%) and progression in 30 patients (21.4%). No significant difference was found among the histoanatomic substaging groups with respect to recurrence rates (*p* = 0.884), whereas the progression rate was markedly higher in the pT1c group compared with the pT1a and pT1b groups (52.2%; *p* < 0.001). In the logistic regression analysis adjusted for age and tumor number, pT1c was independently associated with progression compared with pT1a (OR 6.999; 95% CI 2.328–21.044; *p* = 0.001). Maximum invasion depth and maximum horizontal invasion width were also significantly higher in cases that developed progression and retained their independent associations with progression in the adjusted models. For each 1 mm increase, the OR was 1.209 (95% CI 1.063–1.375; *p* = 0.004) for maximum invasion depth and 1.084 (95% CI 1.007–1.167; *p* = 0.033) for maximum horizontal invasion width. An increasing trend in progression toward T1e was observed in the T1m/T1e classifications, but this did not reach significance in the adjusted models. **Conclusions**: Our findings support that both pT1c invasion extending beyond the muscularis mucosae and continuous quantitative invasion measurements can provide prognostic information in assessing progression risk in pT1 bladder urothelial carcinoma.

## 1. Introduction

Non-muscle-invasive bladder cancer constitutes a substantial proportion of newly diagnosed bladder cancers, and the pT1 stage is defined by tumor invasion into the lamina propria without reaching the muscularis propria. pT1 urothelial carcinomas represent a tumor group that can display marked biological and clinical heterogeneity within the same pathological stage [1,2]. While long-term disease control can be achieved with bladder-sparing approaches in some patients, recurrence or progression to muscle-invasive disease may develop in others; this variable course complicates treatment decisions in pT1 patients [3,4,5]. Therefore, reliably identifying patients at high risk of progression and supporting the existing clinicopathological risk classification with stronger pathological markers represent an important need [1,2].

Current risk classification systems jointly evaluate clinicopathological variables such as tumor stage and grade, tumor number and volume, prior recurrence history, concomitant carcinoma in situ (CIS), and age to predict the likelihood of recurrence and progression in non-muscle-invasive bladder cancer. In the current risk model of the European Association of Urology, tumor stage, WHO 1973 and WHO 2004/2022 grade, concomitant CIS, tumor number, tumor size, and age have been defined as independent determinants of progression, and stratifying patients into low-, intermediate-, high-, and very-high-risk groups based on these parameters has been recommended [6,7]. However, the fact that patients with pT1 tumors of the same stage and similar clinicopathological features can display different clinical courses limits the prognostic discriminative power of the existing risk models at the individual level [1]. Studies summarizing the WHO 5th edition updates likewise emphasize that factors such as tumor grade, size, multifocality, prior recurrence, lymphovascular invasion, and treatment history are important, but that additional histopathological prognostic parameters are needed because of the marked heterogeneity of pT1 disease [6].

One of the most commonly used approaches to define the depth of lamina propria invasion in pT1 bladder urothelial carcinoma in more detail is the substaging system, in which the muscularis mucosae is accepted as a histoanatomic reference point. In this approach, tumor invasion confined above the muscularis mucosae is classified as T1a, invasion reaching the muscularis mucosae as T1b, and invasion extending beyond this layer without reaching the muscularis propria as T1c; when the muscularis mucosae cannot be identified, the vascular plexus within the lamina propria can be used as an auxiliary anatomic landmark [6,8]. Several studies have reported that deeper lamina propria invasion is associated with disease progression in particular, and that T1b/T1c cases can display a less favorable clinical course than T1a cases [1,4,5,8]. However, the most important limitation of this system in routine practice is that the muscularis mucosae shows a discontinuous distribution within the bladder wall and cannot always be reliably identified in transurethral resection specimens. In addition, tissue fragmentation, improper orientation, and thermal artifacts can make it difficult to assess the relationship of the invasion front with the anatomic layers [1,6]. For these reasons, although histoanatomic substaging is prognostically valuable, it has not been established as a single standard method that is applicable and reproducible in all cases.

The difficulties in the applicability of histoanatomic substaging systems have led to the development of micrometric and quantitative approaches aimed at evaluating lamina propria invasion in pT1 tumors more objectively. These methods include the T1m/T1e classification, which distinguishes a single limited invasive focus from more extensive invasion, millimetric measurement of invasion depth, the size of the largest contiguous invasive focus, total invasion length, and the number of invasive foci [4,6,9]. Studies by Van Rhijn et al. and Fransen van de Putte et al. have reported that micrometric substaging carries significant prognostic value in predicting progression and provides stronger discrimination than histoanatomic substaging in some models [4,10]. Budina et al., in turn, showed that invasion depth, the largest invasive focus, total invasion length, and the number of invasive foci were significantly associated with progression [9]. Systematic reviews and meta-analyses likewise support that quantitative substaging approaches can provide clinically meaningful prognostic information, particularly with respect to progression [1,2]. Nevertheless, because of marked differences among the measurement methods and threshold values used, there is still no consensus on the optimal quantitative substaging method.

Although different histoanatomic and quantitative approaches for substaging pT1 bladder urothelial carcinomas have been described in the literature, there is still no consensus on which method most reliably and reproducibly predicts clinical outcomes. In particular, studies directly comparing muscularis mucosae-based classifications with quantitative methods such as micrometric measurement, invasion width, and the number of invasive foci in the same patient group are limited [1,2,4,9]. Therefore, jointly evaluating not only the anatomic level but also the quantitative features of lamina propria invasion in pT1 cases may improve prognostic risk classification. This study aimed to compare the relationship of histoanatomic substaging systems and quantitative lamina propria invasion parameters with recurrence and progression in pT1 bladder urothelial carcinoma, and to evaluate the prognostic value of these approaches.

## 2. Materials and Methods

### 2.1. Study Design and Patient Selection

This study was conducted as a retrospective, single-center cohort study including patients who underwent transurethral resection (TUR) for bladder tumor between **1 January 2020 and 1 December 2025**, and were histopathologically diagnosed with pT1 urothelial carcinoma. Cases in the pathology archive were retrospectively screened, and hematoxylin–eosin-stained slides of patients with a pT1 diagnosis were retrieved from the archive for re-evaluation. The main objective of the study was to investigate the value of histoanatomic and quantitative substaging of lamina propria invasion in predicting tumor recurrence and progression.

Patients in whom lamina propria invasion could be histologically confirmed and the pT1 stage was verified on re-evaluation were included in the study. Patients without recurrence or progression were required to have at least 12 months of clinical follow-up data, whereas patients who developed recurrence or progression within the first 12 months were included regardless of follow-up duration. Cases with missing histologic slides, unavailable clinical or follow-up data, insufficient tumor tissue for reliable assessment of lamina propria invasion, and cases in which the pT1 stage could not be confirmed on re-evaluation were excluded from the study. Specimens in which the muscularis propria and lamina propria smooth muscle fibers could not be reliably distinguished, resulting in staging uncertainty, were also excluded from the analysis. In addition, specimens in which the level or quantitative measurements of lamina propria invasion could not be reliably assessed because of severe cautery artifact, tissue fragmentation, or inadequate orientation were excluded from the relevant analyses.

Muscularis propria was present in all 140 cases forming the final study cohort, and muscularis propria invasion was not detected in any case. After application of the inclusion and exclusion criteria, the final study cohort consisted of 140 patients.

### 2.2. Collection of Clinical and Pathological Data

Patients’ age, sex, and available clinicopathological data were obtained from the hospital records system. Baseline tumor status was classified as primary when the index transurethral resection specimen represented the patient’s initial diagnosis, and as recurrent in patients with a prior history of bladder urothelial tumor. Whether the tumor was solitary or multiple, maximum tumor volume (cc), histologic grade, presence of concomitant carcinoma in situ, and presence or absence of muscularis propria in the transurethral resection specimen were recorded. In cases with available treatment and follow-up data, intravesical treatment history was also evaluated. At the last clinical evaluation, patients’ status was classified as alive without disease, alive with disease, or deceased.

### 2.3. Histopathological Evaluation

The available hematoxylin–eosin-stained sections of all cases included in the study were re-examined. The histopathological sections were independently evaluated by two pathologists **(T.B. and B.S.; see Acknowledgments), who were blinded** to clinical outcomes, including recurrence and **progression. Cases with discordant substaging classifications between the two pathologists were jointly re-reviewed at a multi-head microscope, and a final classification was reached by consensus**. Lamina propria invasion was first confirmed, and the pT1 stage was verified by ensuring the absence of muscularis propria invasion. Histologic grading was performed according to the 2022 World Health Organization classification of urinary tract tumors. Cases classified as low-grade pT1 were separately re-evaluated for both the presence of invasion and histologic grade. In areas of invasive tumor, the anatomic level of lamina propria invasion, the quantitative extent of invasion, and the number of separate invasive foci were systematically assessed. Whether the muscularis mucosae was involved by tumor was additionally recorded as a binary histopathological variable.

For histoanatomic substaging, the muscularis mucosae and its accompanying vascular plexus were used as anatomic references. Tumor invasion confined to the superficial muscularis mucosae was classified as T1a, invasion reaching the muscularis mucosae or observed within this structure as T1b, and invasion extending beyond the muscularis mucosae without reaching the muscularis propria as T1c. This approach was consistent with the histoanatomic system previously used for substaging pT1 urothelial carcinomas [4,5].

Muscularis mucosae could not be directly identified at the tumor invasion front in some specimens; in these instances, assessment was performed taking into account the muscularis mucosae-vascular plexus relationship in the available sections. In these cases, the muscularis mucosae-vascular plexus relationship in adjacent areas was used as an auxiliary anatomic reference.

### 2.4. Quantitative Invasion Assessment

In addition to histoanatomic classification, lamina propria invasion was quantitatively assessed. The maximum invasion depth, maximum horizontal invasion width, and number of invasive foci of the invasive tumor were recorded. Linear measurements were performed in millimeters using a digital imaging platform. Maximum invasion depth was defined as the greatest distance between the most superficial and deepest points of the invasive tumor, measured perpendicular to the mucosal surface. Maximum horizontal invasion width was accepted as the widest diameter of a single continuous invasive focus in the direction parallel to the mucosal surface. Invasive areas separated from one another by tumor-free lamina propria were considered separate foci. In cases with more than one invasive area, the separate tumor foci were reviewed, and the area showing the deepest invasion was used to determine the maximum invasion depth.

Micrometric substaging was assessed using two different approaches. In this assessment, the maximum size of a focus was defined as the larger of that invasive focus’s maximum invasion depth and maximum horizontal invasion width. In the single-focus-based classification, cases with only one invasive focus whose maximum size was 0.5 mm or smaller were classified as T1m; cases with an invasive focus whose maximum size exceeded 0.5 mm or with more than one invasive focus were classified as T1e.

In the three-focus-based classification, cases with at most three separate invasive foci, each with a maximum size of 0.5 mm or smaller, were accepted as T1m. Cases were classified as T1e if any invasive focus exceeded 0.5 mm in size or if there were more than three separate invasive foci.

The use of quantitative invasion assessment was based on previous studies showing that measurements independent of anatomic landmarks can provide prognostic information in pT1 tumors. Budina et al. showed that parameters such as invasion depth, the largest contiguous invasive focus, total invasion length, and the number of invasive foci may be associated with progression [9]. Similarly, it has previously been reported that micrometric substaging systems may serve as an alternative or complementary approach to classic muscularis mucosae-based methods [4,10].

### 2.5. Clinical Outcomes and Follow-Up

The primary clinical outcomes of the study were defined as tumor recurrence and disease progression. Recurrence was defined as histopathological detection of a new bladder urothelial tumor during follow-up after the index pT1 transurethral resection. Progression was defined as the development of urothelial carcinoma showing muscularis propria invasion and/or regional or distant metastatic disease during follow-up. In the pT1 substaging literature, progression has similarly been assessed primarily as progression to muscle-invasive disease [1].

All-cause mortality was evaluated as a secondary outcome. Patients’ final clinical status was recorded as alive without disease, alive with disease, or deceased at the last available follow-up date. In the mortality analyses, patients were evaluated in two groups: alive and deceased.

Patients’ total follow-up duration was recorded in months as the time elapsed from the date of the index pT1 diagnosis to the date of the last clinical evaluation or death. Recurrence and progression were evaluated as binary clinical outcomes according to whether or not they occurred during the follow-up period.

### 2.6. Ethical Approval

The study was conducted in accordance with the principles of the Declaration of Helsinki following approval from the Gazi Yaşargil Training and Research Hospital Ethics Committee (Protocol No. 22; 9 January 2026). Given the retrospective design, only archival materials and existing clinical and follow-up records were used. Patient-identifying information was removed from the analysis dataset.

### 2.7. Statistical Analysis

Statistical analyses were performed using IBM SPSS Statistics version 25.0 (IBM Corp., Armonk, NY, USA). Continuous variables were summarized as mean ± standard deviation, median, 25th–75th percentile, and minimum–maximum values; categorical variables were summarized as number and percentage. The Mann–Whitney U test was used to compare continuous variables between two independent groups. Associations between categorical variables were assessed using the Pearson chi-square test; Fisher’s exact test was used when expected cell counts were low.

Recurrence, progression, and all-cause mortality rates were compared among the muscularis mucosae-based pT1 substaging groups. Pairwise post-hoc comparisons were performed when the overall comparison was significant. To control for the increase in type I error that could result from multiple testing in the pairwise comparisons among the three groups, Bonferroni correction was applied, and the corrected significance threshold was accepted as *p* < 0.0167. A two-sided *p* < 0.05 was accepted as statistically significant for the two-group T1m/T1e comparisons and the other analyses.

The relationship between maximum invasion depth and maximum horizontal invasion width was assessed using Spearman’s rank correlation analysis. Because a high degree of correlation was found between these two quantitative variables (Spearman ρ = 0.904; *p* < 0.001), they were not included together in the multivariable models.

Binary logistic regression analysis was used to evaluate factors independently associated with progression. Progression was coded as the dependent variable, with 0 = absent and 1 = present. To assess the independent effects of different pathological parameters related to lamina propria invasion, each parameter was examined together with age and tumor number in separate multivariable logistic regression models. Tumor number was coded as a categorical variable, as solitary or multiple. Age and tumor number were included in the models as adjustment variables given their clinical relevance. Evaluating each histopathological parameter in a separate model was intended to limit model complexity and the risk of overfitting relative to the available number of progression events. In the histoanatomic substaging analysis, pT1a was used as the reference category, and in the single-focus- and three-focus-based T1m/T1e analyses, T1m was used as the reference category. Maximum invasion depth and maximum horizontal invasion width were modeled as continuous variables, and odds ratios were calculated for each 1 mm increase. Results were reported as odds ratio (OR) and 95% confidence interval (CI). The fit of the logistic regression models was assessed using the Hosmer–Lemeshow test.

## 3. Results

A total of 140 cases of pT1 bladder urothelial carcinoma were included in the study. The mean age of the patients was 65.4 ± 10.9 years, with a median age of 65.0 years and an age range of 33–87 years. Of the cases, 125 (89.3%) were male and 15 (10.7%) were female. According to clinical and macroscopic records, the tumor was reported as solitary in 119 patients (85.0%) and multiple in 21 patients (15.0%). Of the cases, 130 (92.9%) were primary and 10 (7.1%) were recurrent tumors. According to the World Health Organization (WHO) 2022 grading, 131 tumors (93.6%) were high-grade and nine (6.4%) were low-grade. The nine low-grade cases were re-evaluated for both the presence of invasion and histologic grade. Concomitant carcinoma in situ was detected in three cases (2.1%). Muscularis propria was present in 140 cases (100.0%), and propria invasion was not detected in any of them. Muscularis mucosae invasion was present in 51 cases (36.4%) and absent in 89 cases (63.6%). When the number of invasive tumor foci was assessed, 1–4 invasive foci were found in 90 cases (64.3%), 5–9 in 16 cases (11.4%), and 10 or more in 34 cases (24.3%). Based on histoanatomic substaging according to the muscularis mucosae, 89 cases (63.6%) were classified as pT1a, 28 cases (20.0%) as pT1b, and 23 cases (16.4%) as pT1c (Figure 1 and Figure 2). In the single-focus-criterion T1m/T1e classification, 49 cases (35.0%) were T1m and 91 cases (65.0%) were T1e; in the three-focus-criterion T1m/T1e classification, 77 cases (55.0%) were T1m and 63 cases (45.0%) were T1e (Figure 3). Histoanatomic substaging and quantitative invasion parameters could be assessed in all 140 cases. Tumor volume was 6.1 ± 10.4 cc on average with a median of 4.0 cc; maximum invasion depth averaged 2.3 ± 3.7 mm with a median of 0.5 mm and a range of 0.3–23 mm; and maximum horizontal invasion width averaged 3.3 ± 5.6 mm with a median of 0.5 mm and a range of 0.3–36 mm. The median follow-up duration was 24 months, ranging from 12 to 48 months. During follow-up, recurrence developed in 55 patients (39.3%) and progression in 30 patients (21.4%). At the last evaluation, 95 patients (67.9%) were alive without disease, 30 (21.4%) were alive with disease, and 15 (10.7%) were deceased. Deaths were evaluated as all-cause mortality (Table 1).

No significant difference was found among the muscularis mucosae-based pT1 substaging groups with respect to recurrence rates (*p* = 0.884). Recurrence rates were 40.4%, 39.3%, and 34.8% in the pT1a, pT1b, and pT1c groups, respectively. In contrast, progression rates differed significantly among the substaging groups (*p* < 0.001). Progression was observed in 14 patients (15.7%) in the pT1a group, four patients (14.3%) in the pT1b group, and 12 patients (52.2%) in the pT1c group. In the pairwise comparisons using the Bonferroni-corrected significance threshold, the progression rate in the pT1c group was significantly higher than in both the pT1a group (*p* < 0.001) and the pT1b group (*p* = 0.004). No significant difference in progression was found between the pT1a and pT1b groups (*p* = 1.000). All-cause mortality rates also differed significantly among the substaging groups (Fisher’s exact test, *p* = 0.013). The mortality rate was 5.6% in the pT1a group, 14.3% in the pT1b group, and 26.1% in the pT1c group. In the pairwise comparisons using the Bonferroni-corrected significance threshold, the all-cause mortality rate was significantly higher in the pT1c group than in the pT1a group (Fisher’s exact test, *p* = 0.009). In contrast, no significant difference was found between the pT1a and pT1b groups (*p* = 0.215) or between the pT1b and pT1c groups (*p* = 0.316) (Table 2).

The Pearson chi-square test was used for overall comparisons when expected cell counts were adequate, and Fisher’s exact test was used when they were low. Bonferroni correction was applied for pairwise post-hoc comparisons, and the significance level was accepted as *p* < 0.0167. Mortality was evaluated as all-cause death.

When quantitative lamina propria invasion parameters were evaluated with respect to recurrence, no significant difference in maximum invasion depth was found between cases with and without recurrence; the median maximum invasion depth was 0.5 mm in both groups [0.5 (0.4–3.0) mm vs. 0.5 (0.4–2.0) mm; *p* = 0.910]. Similarly, maximum horizontal invasion width did not differ significantly according to recurrence status [0.5 (0.4–4.0) mm in cases without recurrence vs. 0.5 (0.4–3.0) mm in those with recurrence; *p* = 0.598]. The distribution of invasive focus number categories also did not differ significantly according to recurrence status (*p* = 0.803). In contrast, maximum invasion depth was significantly higher in cases that developed progression. The median maximum invasion depth was 0.5 mm (0.4–2.0) in cases without progression and 3.0 mm (0.4–5.0) in those with progression (*p* = 0.002). Maximum horizontal invasion width was also significantly higher in cases with progression [4.0 (0.4–7.0) mm vs. 0.5 (0.4–2.0) mm; *p* = 0.033]. The distribution of invasive focus number categories did not differ significantly according to progression status (*p* = 0.203). When evaluated with respect to all-cause mortality, neither maximum invasion depth nor maximum horizontal invasion width differed significantly between deceased and surviving patients. Maximum invasion depth was 3.0 (0.4–5.0) mm in deceased patients and 0.5 (0.4–2.0) mm in survivors (*p* = 0.128), while maximum horizontal invasion width was 4.0 (0.4–12.0) mm and 0.5 (0.4–3.0) mm, respectively (*p* = 0.182). In the distribution of invasive focus number categories according to all-cause mortality status, the proportion of the ≥10-focus group among deceased patients appeared higher, but this difference did not reach statistical significance (Fisher’s exact test, *p* = 0.067) (Table 3).

In the single-focus-based T1m/T1e classification, the recurrence rate was 35.2% in the T1e group and 46.9% in the T1m group, with no significant difference between the groups (*p* = 0.174). In contrast, progression developed in 25 of 91 patients (27.5%) in the T1e group and in five of 49 patients (10.2%) in the T1m group, and the progression rate was significantly higher in the T1e group (*p* = 0.018). The all-cause mortality rate was 13.2% and 6.1% in the T1e and T1m groups, respectively, but the difference was not statistically significant (*p* = 0.197). In the three-focus-based T1m/T1e classification, recurrence rates were 38.1% and 40.3% in the T1e and T1m groups, respectively, with no significant difference between the groups (*p* = 0.794). Although progression occurred in 18 patients (28.6%) in the T1e group and 12 patients (15.6%) in the T1m group, this difference did not reach statistical significance (*p* = 0.062). Similarly, all-cause mortality occurred in 14.3% of the T1e group and 7.8% of the T1m group, and the difference between the groups was not significant (*p* = 0.217) (Table 4).

Separate multivariable logistic regression models adjusted for age and tumor number were constructed to evaluate the lamina propria invasion parameters independently associated with progression. Histoanatomic substaging overall was independently associated with progression (*p* = 0.001). While the odds of progression in the pT1b group did not differ significantly from the pT1a group (OR = 0.710; 95% CI: 0.194–2.599; *p* = 0.605), the odds of progression in the pT1c group were approximately seven times higher than in the pT1a group (OR = 6.999; 95% CI: 2.328–21.044; *p* = 0.001). Maximum invasion depth was independently associated with progression after adjustment for age and tumor number (OR = 1.209; 95% CI: 1.063–1.375; *p* = 0.004). Each 1 mm increase in maximum invasion depth was associated with an approximately 21% increase in the odds of progression. Maximum horizontal invasion width was also independently associated with progression in the adjusted model (OR = 1.084; 95% CI: 1.007–1.167; *p* = 0.033). Each 1 mm increase in maximum horizontal invasion width was associated with an approximately 8% increase in the odds of progression. In the single-focus-based T1e classification, the odds of progression were higher than in T1m, but the result did not reach statistical significance (OR = 2.683; 95% CI: 0.920–7.822; *p* = 0.071). Similarly, higher odds of progression were observed in the three-focus-based T1e classification, but the association was not significant (OR = 2.210; 95% CI: 0.920–5.312; *p* = 0.076) (Table 5).

**Each lamina propria invasion parameter was evaluated together with age and tumor number in a separate multivariable binary logistic regression model. pT1a and T1m were used as reference categories. Because of the high correlation between maximum invasion depth and maximum horizontal invasion width (Spearman ρ = 0.904;** ***p*** **< 0.001), these variables were not included together in the same model. The Hosmer–Lemeshow test indicated adequate fit for all models (histoanatomic substaging,** ***p*** **= 0.405; maximum invasion depth,** ***p*** **= 0.853; maximum horizontal invasion width,** ***p*** **= 0.190; single-focus T1m/T1e,** ***p*** **= 0.824; three-focus T1m/T1e,** ***p*** **= 0.427).**

## 4. Discussion

In this study, evaluation of lamina propria invasion in pT1 bladder urothelial carcinoma using different substaging approaches showed that both the histoanatomic level and the quantitative extent of invasion can provide prognostic information in determining progression risk. In the muscularis mucosae-based histoanatomic substaging system, the progression rate was markedly higher in pT1c cases, and this association remained independent after adjustment for age and tumor number. Similarly, maximum invasion depth and maximum horizontal invasion width were higher in cases that developed progression, and both parameters showed an independent association with progression in the adjusted models. In contrast, although higher progression rates were observed in the T1e groups in the T1m/T1e-based categorical micrometric classifications, these associations did not reach statistical significance in the multivariable analyses. In addition, none of the substaging methods evaluated provided a clear distinction with respect to recurrence. These results suggest that both the anatomic depth of invasion and continuous quantitative measurements can provide complementary information in assessing progression risk in pT1 tumors.

With respect to histoanatomic substaging, our findings are consistent with previous studies showing that invasion extending beyond the muscularis mucosae in particular is associated with a higher risk of progression. Orsola et al. reported that in high-grade pT1 tumors, the risk of progression was significantly higher in the T1b/T1c group than in the T1a group, and that the depth of lamina propria invasion carried independent prognostic value for progression [5]. Similarly, Grobet-Jeandin et al. showed that pT1 substaging was independently associated with progression and that a less favorable clinical course was observed in the T1b and T1c groups compared with T1a [11]. More recent studies have reported similar results. In the study by Sato et al. evaluating en bloc resection specimens, muscularis mucosae-based substaging was found to be an independent prognostic factor for progression, whereas the micrometric approach did not show independent significance [12]. Kläger et al., in a series directly comparing anatomic and micrometric substaging methods, reported that only anatomic landmark-based substaging was independently associated with progression to T2 [13]. In the large high-grade T1 cohort of Finati et al., muscularis mucosae invasion was found to be associated with upstaging to muscle-invasive disease at repeat transurethral resection [14]. The finding in our study that the pT1c group remained associated with approximately seven-fold higher odds of progression compared with pT1a after adjustment for age and tumor number supports that invasion extending beyond the muscularis mucosae may be a valuable histopathological indicator for identifying a subgroup with higher progression risk among pT1 tumors.

One of the notable findings of our study was that the substaging methods showed different relationships with recurrence and progression. While recurrence rates were similar among the histoanatomic substaging groups, progression was markedly increased, particularly in the pT1c group. This dissociation is consistent with the previous literature. Grobet-Jeandin et al. reported that pT1 substaging did not show a significant association with recurrence, whereas it was independently associated with progression and overall survival [11]. Similarly, in the series of Lee et al., the recurrence-free interval did not differ among the substaging groups, whereas deeper lamina propria invasion was associated with a shorter progression-free interval and higher progression rates [8]. In the studies of Sato et al. and Kläger et al. as well, anatomic substaging was found to be associated with progression, but a similar prognostic distinction could not be demonstrated for recurrence [12,13]. A recent meta-analysis likewise reported that muscularis mucosae invasion was significantly associated with progression, whereas an association with recurrence could not be statistically demonstrated [2]. These findings suggest that the anatomic depth of lamina propria invasion may reflect the potential for progression to muscle-invasive disease more than the tendency of the tumor to recur. Recurrence, on the other hand, should be considered to be more strongly influenced by additional clinical factors such as prior tumor history, multifocality, and intravesical treatment.

Quantitative invasion measurements also yielded significant results with respect to progression. Maximum invasion depth and maximum horizontal invasion width were higher in cases that developed progression, and both parameters retained their independent association in models adjusted for age and tumor number. Each 1 mm increase in maximum invasion depth was associated with an approximately 21% increase in the odds of progression (OR = 1.209; 95% CI: 1.063–1.375; *p* = 0.004), whereas each 1 mm increase in maximum horizontal invasion width was associated with an approximately 8% increase in the odds of progression (OR = 1.084; 95% CI: 1.007–1.167; *p* = 0.033). Budina et al. also reported that invasion depth, the largest contiguous invasive focus, total invasion length, and the number of invasive foci were associated with progression, and that some of these parameters retained their significance in multivariable analyses [9]. In contrast, in the study by Kläger et al., ALLICA measurements and other micrometric classifications did not show a significant association with progression or recurrence, and only anatomic landmark-based substaging carried independent prognostic value [13]. These differences among studies may be related to the measurement axis, how separate invasive foci are assessed, the threshold values used, and issues of fragmentation and orientation in transurethral resection specimens. In addition, the lack of standardization of the micrometric cutoff values used across different studies and anatomic variability in lamina propria thickness may limit the generalizability of quantitative methods [9].

Nevertheless, it is notable that although maximum invasion depth and horizontal invasion width, evaluated as continuous variables in our study, showed an independent prognostic association, the categorical micrometric classifications in the T1m/T1e format lost their significance in the adjusted analyses. Although the progression rate in the single-focus-based assessment was higher in the T1e group than in the T1m group, the association remained at the borderline of statistical significance after adjustment for age and tumor number; a similar trend was observed in the three-focus-based classification, but independent significance could not be demonstrated. Van Rhijn et al. reported that the T1m/T1e system carried prognostic value for progression and disease-specific survival [4], whereas in the studies of Sato et al. and Kläger et al., categorical micrometric systems did not show a similar independent prognostic performance [12,13]. Although recent meta-analyses have reported that the T1e classification is associated with an increased risk of progression, variability in the threshold values and definitions of invasive foci used across studies makes standardization difficult [2]. Our findings suggest that evaluating invasion as a continuous quantitative variable may provide prognostic information that is not fully captured by categorical classifications based on predetermined thresholds.

The applicability and reproducibility of substaging methods in routine pathology practice are as important as their prognostic performance. The main limitations of the muscularis mucosae-based histoanatomic system are fragmentation of transurethral resection specimens, cautery artifact, inadequate orientation, and the inability to reliably identify the muscularis mucosae in every specimen. Nevertheless, in the series of Grobet-Jeandin et al., substaging could be performed in approximately 90% of cases, and the initial interobserver agreement between two pathologists was reported as 89% [11]. Kläger et al. found the applicability of the anatomic method to be generally acceptable, but showed that fragmentation and tangential sections make assessment more difficult, and that in micrometric methods, the identification of invasive foci and the choice of measurement axis can lead to interobserver variability [13]. The fact that muscularis mucosae-vascular plexus structures could be assessed in all cases in the en bloc resection specimens of Sato et al. suggests that preserving tissue integrity and orientation may improve the applicability of substaging [12]. Therefore, the performance of substaging methods depends not only on the classification system used but also on the resection technique, specimen quality, and standardization of pathological assessment. Although the World Health Organization and current international recommendations support reporting pT1 substaging, there is not yet a common approach regarding which method should be preferred as the standard.

**An important clinical question is whether these substaging parameters improve risk prediction beyond established clinical risk models, such as the European Association of Urology (EAU) prognostic risk groups for non-muscle-invasive bladder cancer [7]. Our study was not designed to answer this question directly. Because of the limited number of progression events (** ***n*** **= 30), the multivariable models were adjusted only for age and tumor number, and not all components of the EAU risk model could be included. In addition, the incremental predictive value of the substaging parameters was not assessed with measures such as the change in the area under the receiver operating characteristic curve or the concordance index, net reclassification improvement, or decision curve analysis. Therefore, our findings show that pT1c status and quantitative invasion measurements are associated with progression independently of age and tumor number, but they do not demonstrate that these parameters improve risk prediction beyond existing clinical models. Because all patients in our cohort had pT1 tumors, most would already fall into the higher-risk categories of current models, and the potential clinical value of substaging may lie in further stratifying progression risk within these categories, for example in selecting candidates for early radical cystectomy versus bladder-sparing management. This hypothesis should be tested in larger, multicenter cohorts with time-to-event data, in which substaging parameters are added to established risk models and the resulting changes in discrimination and calibration are formally evaluated.**

One of the strengths of our study is that lamina propria invasion in pT1 urothelial carcinoma was evaluated using multiple methods in the same patient cohort. Histoanatomic substaging, maximum invasion depth, maximum horizontal invasion width, number of invasive foci, and T1m/T1e-based classifications were compared within the same dataset. In addition, examining histoanatomic substaging and continuous quantitative measurements in separate multivariable models using the same adjustment variables allowed evaluation of the independent associations of the different approaches with progression. The re-review of all histologic slides and the recording of quantitative measurements in millimeters are also aspects that support standardization of the morphological assessment.

This study has several limitations. The retrospective, single-center design limits the generalizability of the results. The number of patients who developed progression being limited to 30 reduced the number of variables that could be evaluated in the multivariable models, and the low frequency of some clinicopathological variables, such as concomitant carcinoma in situ and low-grade tumors, made reliable modeling of these factors difficult. Because event dates were not available, time-to-event survival analyses could not be performed for recurrence and progression, and outcomes were evaluated as binary variables. Because mortality was recorded as all-cause mortality, the mortality data cannot be interpreted as cancer-specific mortality. Fragmentation, cautery artifact, and loss of orientation in transurethral resection specimens are other potential limitations that could affect both anatomic and quantitative assessments. In addition, because of the high correlation between maximum invasion depth and maximum horizontal invasion width, these two variables could not be evaluated together in the same multivariable model, and their fully independent prognostic contributions could not be determined. It should also be considered that, because intravesical treatment data were not complete for the entire cohort, the possible effect of treatment on progression could not be fully controlled for in the multivariable models.

## 5. Conclusions

In conclusion, the histoanatomic approach to substaging lamina propria invasion in pT1 bladder urothelial carcinoma, particularly pT1c invasion extending beyond the muscularis mucosae, showed an independent and marked association with progression. In addition, maximum invasion depth and maximum horizontal invasion width were also independently associated with progression in multivariable analyses adjusted for age and tumor number. In contrast, although the T1m/T1e-based categorical micrometric classifications showed certain trends with respect to progression, they did not reach independent significance in the adjusted models. The limited performance of the substaging methods in predicting recurrence suggests that these approaches may reflect the tendency to progress to muscle-invasive disease more than the tendency of the tumor to recur. These findings suggest that both muscularis mucosae-based histoanatomic substaging and continuous quantitative invasion measurements can contribute to clinical risk assessment in pT1 tumors. Nevertheless, larger, multicenter, prospective studies using standardized definitions are needed to determine the optimal substaging approach and to clarify the clinical utility of the different methods.

## Figures and Tables

**Figure 1:**
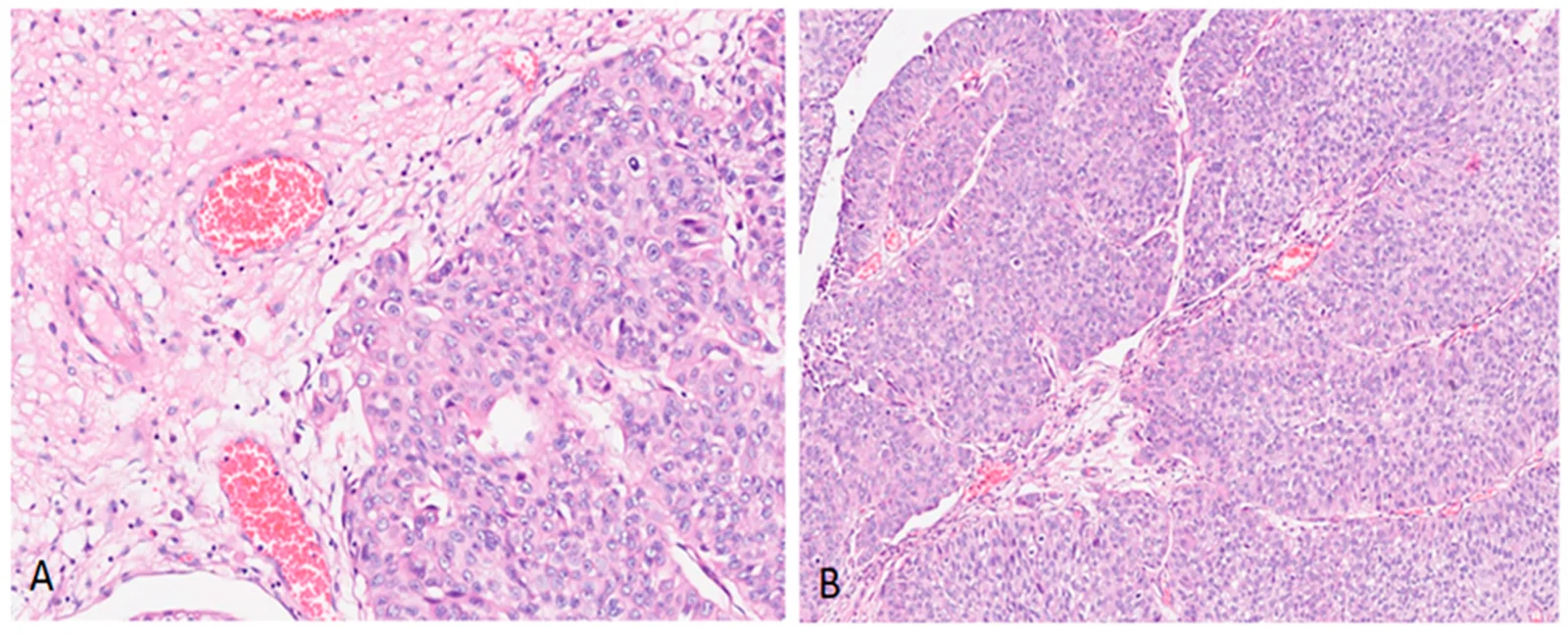
Different morphological appearances of pT1a urothelial carcinoma. (**A**) A small, irregular focus of lamina propria invasion. (**B**) Broader, confluent invasive tumor nests within the lamina propria. Both examples are classified as pT1a on the basis of invasion depth, despite the difference in the extent of invasion. Hematoxylin and eosin.

**Figure 2:**
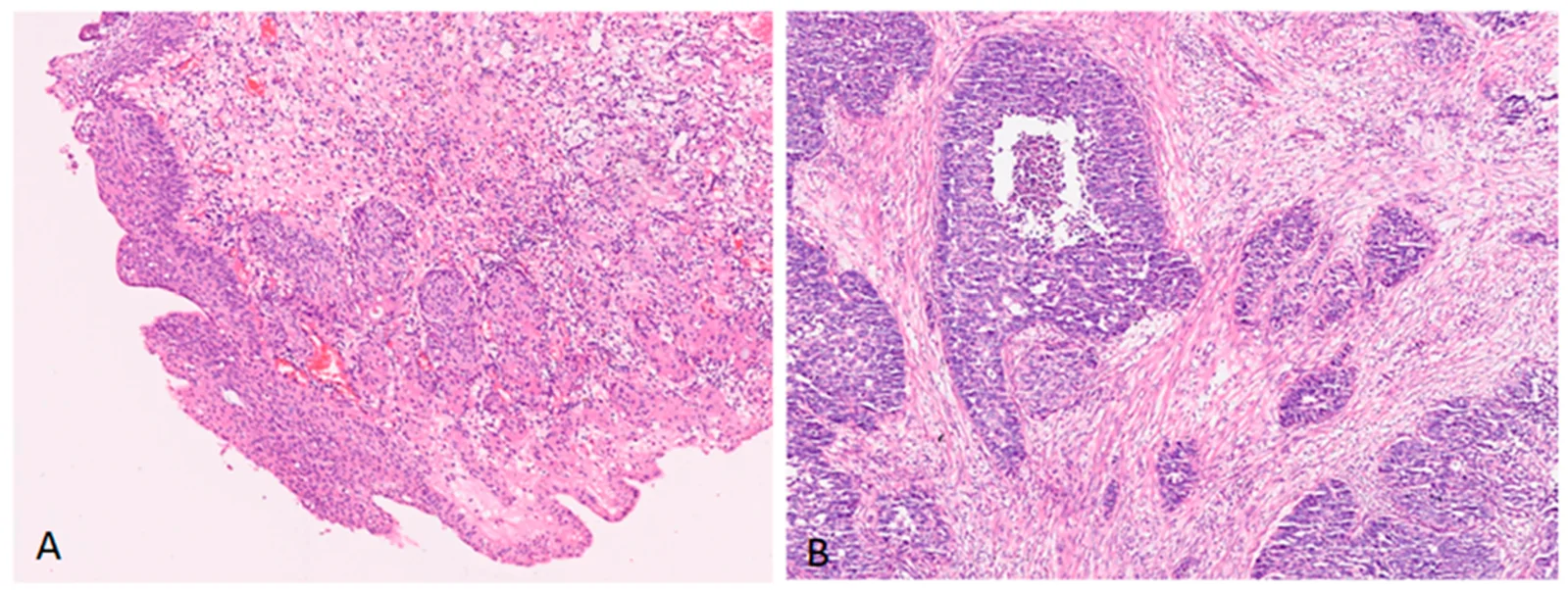
Representative examples of histoanatomic pT1 substaging in urothelial carcinoma. (**A**) pT1b: The muscularis mucosae could not be identified; the lamina propria vascular plexus was used as the anatomic landmark to assess invasion depth. (**B**) pT1c: Tumor nests extend beyond the muscularis mucosae into the deeper lamina propria. Hematoxylin and eosin.

**Figure 3:**
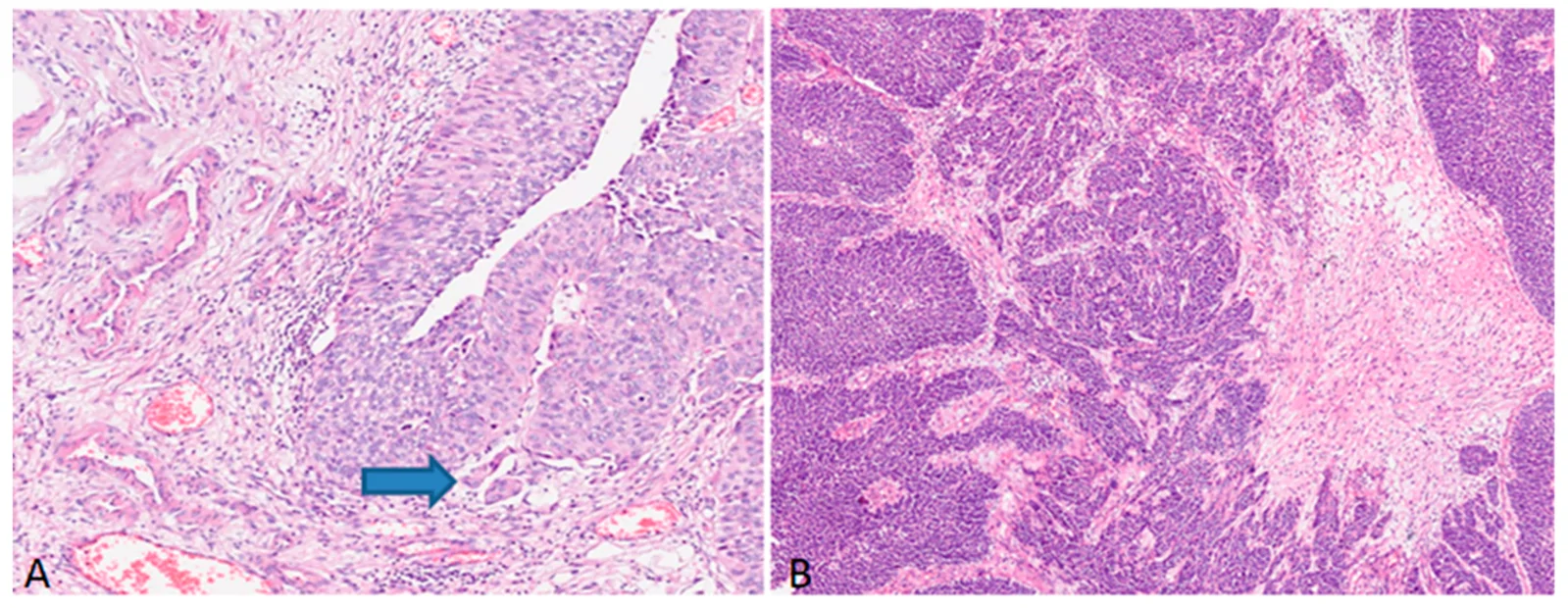
Representative histological images of pT1 urothelial carcinoma. (**A**) pT1m, showing a single focus of lamina propria invasion (arrow). (**B**) pT1e, showing multifocal and extensive lamina propria invasion. Hematoxylin and eosin.

**Table 1:** Clinicopathological characteristics of the study group and histopathological findings related to lamina propria invasion.

		*n*	%
Sex	Female	15	10.7
	Male	125	89.3
Tumor number	Solitary	119	85.0
	Multiple	21	15.0
Tumor status	Primary	130	92.9
	Recurrent	10	7.1
WHO 2022 grade	Low	9	6.4
	High	131	93.6
Carcinoma in situ	Absent	137	97.9
	Present	3	2.1
pT1		140	100.0
Muscularis propria in the specimen	Present	140	100.0
Muscularis propria invasion	Absent	140	100.0
Muscularis mucosae invasion	Absent	89	63.6
	Present	51	36.4
Number of invasive foci	1–4	90	64.3
	5–9	16	11.4
	≥10	34	24.3
Recurrence	No	85	60.7
	Yes	55	39.3
Progression	No	110	78.6
	Yes	30	21.4
Patient status	Alive without disease	95	67.9
	Alive with disease	30	21.4
	Deceased	15	10.7
Substaging according to the muscularis mucosae	pT1a	89	63.6
	pT1b	28	20.0
	pT1c	23	16.4
Single-focus-criterion T1m/T1e	T1e	91	65.0
	T1m	49	35.0
Three-focus-criterion T1m/T1e	T1e	63	45.0
	T1m	77	55.0
	**Minimum–maximum**	**Mean ± SD**	**Median**
Age	33–87	65.4 ± 10.9	65.0
Tumor volume (cc)	1–100	6.1 ± 10.4	4.0
Maximum invasion depth (mm)	0.3–23	2.3 ± 3.7	0.5
Maximum horizontal invasion width (mm)	0.3–36	3.3 ± 5.6	0.5
Follow-up duration (months)	12–48	29.1 ± 11.6	24.0

**Table 2:** Comparison of recurrence, progression, and all-cause mortality according to muscularis mucosae-based pT1 substaging groups.

			Substaging			*p*
			pT1a	pT1b	pT1c	
Recurrence	No	*n*	53	17	15	0.884
		%	59.6%	60.7%	65.2%	
	Yes	*n*	36	11	8	
		%	40.4%	39.3%	34.8%	
Progression	No	*n*	75	24	11	**<0.001**
		%	84.3%	85.7%	47.8%	
	Yes	*n*	14	4	12	
		%	15.7%	14.3%	52.2%	
Mortality	Alive	*n*	84	24	17	**0.013**
		%	94.4%	85.7%	73.9%	
	Deceased	*n*	5	4	6	
		%	5.6%	14.3%	26.1%	

**Table 3:** Association of quantitative lamina propria invasion parameters with recurrence, progression, and all-cause mortality.

Variable	No Recurrence (*n* = 85)	Recurrence (*n* = 55)	*p*	No Progression (*n* = 110)	Progression (*n* = 30)	*p*	Alive (*n* = 125)	Deceased (*n* = 15)	*p*
**Max invasion depth, mm**	0.5 (0.4–3.0)	0.5 (0.4–2.0)	0.910	0.5 (0.4–2.0)	3.0 (0.4–5.0)	**0.002**	0.5 (0.4–2.0)	3.0 (0.4–5.0)	0.128
**Max horizontal invasion width, mm**	0.5 (0.4–4.0)	0.5 (0.4–3.0)	0.598	0.5 (0.4–2.0)	4.0 (0.4–7.0)	**0.033**	0.5 (0.4–3.0)	4.0 (0.4–12.0)	0.182
**Number of invasive foci**	***n*** **(%)**	***n*** **(%)**		***n*** **(%)**	***n*** **(%)**		***n*** **(%)**	***n*** **(%)**	
1–4	56 (65.9)	34 (61.8)	0.803	74 (67.3)	16 (53.3)	0.203	84 (67.2)	6 (40.0)	0.067 *
5–9	10 (11.8)	6 (10.9)		13 (11.8)	3 (10.0)		14 (11.2)	2 (13.3)	
≥10	19 (22.4)	15 (27.3)		23 (20.9)	11 (36.7)		27 (21.6)	7 (46.7)	

Data are presented as median (25th–75th percentile) or *n* (%). The Mann–Whitney U test was used to compare continuous variables, and the Pearson chi-square test was used for categorical variables. * Fisher’s exact test was used because of low expected cell counts.

**Table 4:** Association of single-focus- and three-focus-based T1m/T1e classifications with recurrence, progression, and all-cause mortality.

Classification	Recurrence, *n* (%)	*p*	Progression, *n* (%)	*p*	Deceased, *n* (%)	*p*
**Single-focus T1m/T1e**						
T1e (*n* = 91)	32 (35.2)	0.174	25 (27.5)	**0.018**	12 (13.2)	0.197
T1m (*n* = 49)	23 (46.9)		5 (10.2)		3 (6.1)	
**Three-focus T1m/T1e**						
T1e (*n* = 63)	24 (38.1)	0.794	18 (28.6)	0.062	9 (14.3)	0.217
T1m (*n* = 77)	31 (40.3)		12 (15.6)		6 (7.8)	

Data are presented as *n* (%). Percentages were calculated based on the number of patients in the corresponding T1m/T1e group. The Pearson chi-square test was used to compare the groups. Statistical significance was accepted as *p* < 0.05.

**Table 5:** Association of lamina propria invasion parameters with progression in logistic regression analyses adjusted for age and tumor number.

Pathological Parameter	Adjusted OR	95% CI	*p*
**Histoanatomic substaging, overall**			**0.001**
pT1b vs. pT1a	0.710	0.194–2.599	0.605
pT1c vs. pT1a	6.999	2.328–21.044	**0.001**
**Maximum invasion depth, per 1 mm increase**	1.209	1.063–1.375	**0.004**
**Maximum horizontal invasion width, per 1 mm increase**	1.084	1.007–1.167	**0.033**
**Single-focus T1e vs. T1m**	2.683	0.920–7.822	0.071
**Three-focus T1e vs. T1m**	2.210	0.920–5.312	0.076

## Data Availability

The data supporting the findings of this study are available from the corresponding author upon reasonable request. The data are not publicly available because they contain information that could compromise patient privacy.

## References

[1] Parizi M.K., Enikeev D., Glybochko P.V., Seebacher V., Janisch F., Fajkovic H., Chłosta P.L., Shariat S.F. (2020). Prognostic value of T1 substaging on oncological outcomes in patients with non-muscle-invasive bladder urothelial carcinoma: A systematic literature review and meta-analysis. World J. Urol..

[2] Piccolini A., Moretto S., Fasulo V., Uleri A., Contieri R., Finocchiaro A., Cella L., Dagnino F., Avolio P.P., Saita A. (2026). Prognostic Role of Different T1 Substaging Systems on Recurrence and Progression in Non-muscle-invasive Bladder Cancer: A Systematic Review and Meta-analysis. Eur. Urol. Oncol..

[3] Breyer J., Wirtz R.M., Otto W., Erben P., Kriegmair M.C., Stoehr R., Eckstein M., Eidt S., Denzinger S., Burger M. (2017). In stage pT1 non-muscle-invasive bladder cancer (NMIBC), high KRT20 and low KRT5 mRNA expression identify the luminal subtype and predict recurrence and survival. Virchows Arch..

[4] Van Rhijn B.W., Van der Kwast T.H., Alkhateeb S.S., Fleshner N.E., Van Leenders G.J., Bostrom P.J., van der Aa M.N., Kakiashvili D.M., Bangma C.H., Jewett M.A. (2012). A new and highly prognostic system to discern T1 bladder cancer substage. Eur. Urol..

[5] Orsola A., Trias I., Raventós C., Español I., Cecchini L., Búcar S., Salinas D., Orsola I. (2005). Initial high-grade T1 urothelial cell carcinoma: Feasibility and prognostic significance of lamina propria invasion microstaging (T1a/b/c) in BCG-treated and BCG-non-treated patients. Eur. Urol..

[6] Guo C.C., Shen S.S., Czerniak B. (2023). Recent Advances in the Classification of Bladder Cancer—Updates from the 5th Edition of the World Health Organization Classification of the Urinary and Male Genital Tumors. Bladder Cancer.

[7] Gontero P., Birtle A., Compérat E., Dominguez J.L., Liedberg F., Mariappan P. (2024). EAU Guidelines on Non-muscle-invasive Bladder Cancer (TaT1 and CIS). https://d56bochluxqnz.cloudfront.net/documents/full-guideline/EAU-Guidelines-on-Non-muscle-invasive-Bladder-Cancer-2026.pdf.

[8] Lee J.Y., Joo H.J., Cho D.S., Kim S.I., Ahn H.S., Kim S.J. (2012). Prognostic significance of substaging according to the depth of lamina propria invasion in primary T1 transitional cell carcinoma of the bladder. Korean J. Urol..

[9] Budina A., Farahani S.J., Lal P., Nayak A. (2022). Subcategorization of T1 Bladder Cancer on Biopsy and Transurethral Resection Specimens for Predicting Progression. Arch. Pathol. Lab. Med..

[10] van de Putte E.E.F., Otto W., Hartmann A., Bertz S., Mayr R., Bründl J., Breyer J., Manach Q., Compérat E.M., Boormans J.L. (2018). Metric substage according to micro and extensive lamina propria invasion improves prognostics in T1 bladder cancer. Urol. Oncol. Semin. Orig. Investig..

[11] Grobet-Jeandin E., Wirth G.J., Benamran D., Dupont A., Tille J.C., Iselin C.E. (2022). Substaging of pT1 Urothelial Bladder Carcinoma Predicts Tumor Progression and Overall Survival. Urol. Int..

[12] Sato S., Yanagisawa T., Miki J., Hayashida Y., Okada Y., Iwatani K., Matsukawa A., Kimura T., Egawa S., Shimoda M. (2024). pT1 Subclassification Predicts Progression-Free Survival in En Bloc Resection of Bladder Tumor Specimens. Arch. Pathol. Lab. Med..

[13] Kläger J., Koeller M.C., Oszwald A., Wasinger G., D’Andrea D., Compérat E. (2025). A single-center retrospective comparison of pT1 substaging methods in bladder cancer. Virchows Arch..

[14] Finati M., Fanelli A., Cinelli F., Schiavone N., Falagario U.G., Ricapito A., D’aLtilia N., Naspro R., Porreca A., Crocetto F. (2025). Oncological outcomes and prognostic implications of T1 histo-anatomic substaging in the management of high-grade non-muscle invasive bladder cancer: Results from a large single centre series. World J. Urol..

